# Brain Abscess Secondary to a Dental Infection

**DOI:** 10.1155/2020/3248174

**Published:** 2020-02-06

**Authors:** Léonor Costa Mendes, Frédéric Vaysse, Delphine Maret

**Affiliations:** ^1^Centre Hospitalier Universitaire, UFR Odontologie, Université Paul Sabatier, Toulouse, France; ^2^Centre Hospitalier Universitaire, Laboratoire Anthropologie Moléculaire et Imagerie de Synthèse, UFR Odontologie, Université Paul Sabatier, Toulouse, France

## Abstract

The risk of a brain abscess is a complication of odontogenic infection that is rarely considered by physicians and little spoken of, yet treating dental infections may avoid a potentially life-threatening condition. We report a case of 7-year-old boy with a brain abscess secondary to a dental infection. He was immediately taken to the operating theatre for drainage and cleaning of the abscess. A dental examination revealed root abscesses on temporary molars, which were extracted under general anaesthetic. Two months after his admission, the child was switched to oral antibiotherapy and could return home. A brain abscess represents a life-threatening disease. Childhood brain abscess is uncommon but may be encountered by all physicians and students as a clinical emergency. It is indispensable that physicians finding symptoms similar to those in this case study refer the patient for emergency care and that possible dental foci of infection be assessed, whether or not the patient is being followed for dental care.

## 1. Introduction

A brain abscess (BA) is a clinical emergency because of the significant risk of long-term morbidity and mortality associated with it, despite medical advances [1, 2]. Whatever the patient's age, a brain abscess requires medical and surgical treatment [3]. Such abscesses correspond to a focal infection in the brain parenchyma, characterized by localized oedema and inflammation causing a well circumscribed accumulation of pus [4]. Although childhood brain abscess is uncommon, all physicians and students may encounter it.

The most widespread primary sources of brain infection are infectious endocarditis, osteomyelitis, bacteraemia, and lung, abdominal, pelvic, skin, or ENT diseases [1–4]. The risk of a brain abscess is a complication of odontogenic infection that is rarely considered, yet treating dental infections may avoid a potentially life-threatening condition.

## 2. Case Report

**Figure 1:**
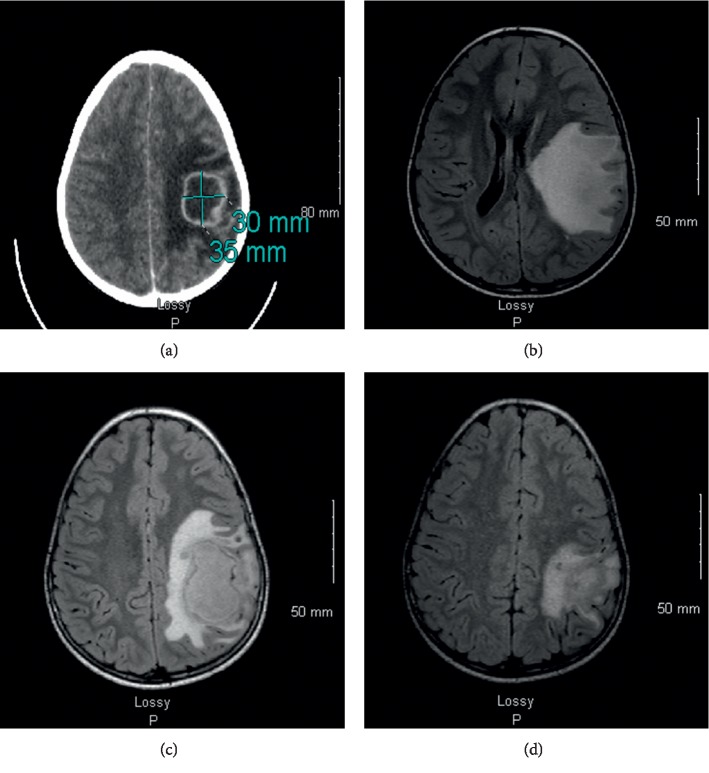
(a) Left fronto-parietal abscess revealed by a brain scan. (b) Axial view of the magnetic resonance imaging reveals a 45 × 52 mm abscess in the left front-temporal lobe of the brain (T0). (c) Axial view of the magnetic resonance imaging reveals an increase in the volume of the abscess, justifying a second drainage (*T* + 23 days). (d) Axial view of the magnetic resonance imaging shows a decrease in the abscess relative to the previous image (Figure 1(c)) (*T* + 2 months).

**Figure 2:**
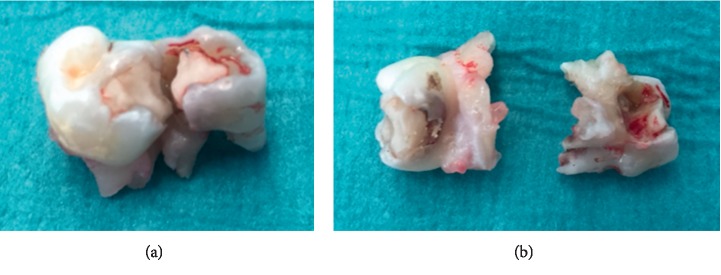
Temporary molars 64 and 65 presented root abscesses and were extracted under general anaesthetic thirteen days after arrival at the emergency department.

A previously healthy boy aged 7 years and 11 months was brought to the hospital emergency department suffering from persistent headache and vomiting. He had had a temperature of 38°C for a week and presented a motor deficit of the right arm associated with paresthesiae. A brain CT scan revealed a left fronto-parietal abscess (Figure 1(a)). Neither his medical history nor the clinical examination provided evidence of ENT (Ear, Nose, and Throat) infection. His mother reported that he had had dental treatment for decay in the left temporal molars three weeks earlier. A brain MRI scan showed a left fronto-parietal lesion of approximately 45 × 52 mm with a right lateral deviation of the median line (Figure 1(b)). He was immediately taken to the operating theatre for drainage and cleaning of the abscess. Intraoperative bacteriological specimens were taken, and broad spectrum antibiotherapy was set up using cefotaxime and metronidazole. Corticotherapy (Solumedrol®) was started the following day and continued for six days. Targeted antibiotherapy was set up when the bacteriological analysis results were available. Direct examination of the bacteriological culture found Gram + cocci in chains, and aerobic and anaerobic culture revealed the presence of *Streptococcus intermedius*. Two days after surgery, the child was apyretic, with normal heart rate and blood pressure but persistence of a motor deficit of the right arm. A dental examination revealed root abscesses on temporary molars 64 and 65, which were extracted under general anaesthetic thirteen days after the child's arrival at the emergency department (Figure 2). Initial dental treatment was insufficient to manage a potential infection that later became acute.

Twenty-three days after the initial drainage, a control MRI performed because of headaches and vomiting imaged an increase in the volume of the abscess, justifying a second drainage (Figure 1(c)). Bacteriological culture showed the specimen to be sterile. Two months after the first operation, the neurological situation had evolved favourably, the deficit affecting only fine motor skills of the right hand. The brain MRI scan showed a marked decrease in the abscess relative to the previous image (Figure 1(d)). Two months after his admission, the child was switched to oral antibiotherapy and could return home. Follow-up with control brain MRI was planned at the Children's Hospital at 3 weeks, with a view to stopping the antibiotic treatment.

## 3. Discussion

The multiplicity of possible sources of infection and the diversity of oral bacteria mean that the implication of dental infection in brain abscesses is indicated by the failure to find any other infection source, the presence of oral microflora in the abscess microbiological spectrum, and clinical and radiographic signs of dental infection [5]. Frequently associated with brain abscess in adults and children, *Streptococcus intermedius* is a commensal bacterium found in the oral cavity and gastrointestinal tract [6, 7]. The discovery of this organism in bacteriological cultures should lead the clinician to consider an oral and dental aetiology. In this case report, the delay between neurosurgical and dental procedures is explained by the lack of knowledge of this dental aetiology. Fortunately, the patient made a full recovery without sequelae, but morbidity of brain abscesses can reach 53% and mortality 16% of cases [7].
